# Emerging Anti-Atherosclerotic Therapies

**DOI:** 10.3390/ijms222212109

**Published:** 2021-11-09

**Authors:** Anna Gluba-Brzózka, Beata Franczyk, Magdalena Rysz-Górzyńska, Janusz Ławiński, Jacek Rysz

**Affiliations:** 1 Department of Nephrology, Hypertension and Family Medicine, Medical University of Lodz, 90-549 Lodz, Poland; beata.franczyk-skora@umed.lodz.pl (B.F.); jacek.rysz@umed.lodz.pl (J.R.); 2 Department of Ophthalmology and Visual Rehabilitation, Medical University of Lodz, 90-549 Lodz, Poland; mrs-89@o2.pl; 3 Department of Urology, Institute of Medical Sciences, Medical College of Rzeszow University, 35-055 Rzeszow, Poland; janlaw@wp.pl

**Keywords:** atherosclerosis, cyclodextrins, protein kinase inhibitors, colchicine, inhibitors of p38 mitogen-activated protein kinase (MAPK), lipid dicarbonyl scavengers, monoclonal antibody targeting interleukin-1β, P-selectin inhibitors

## Abstract

Cardiovascular disease (CAD) is the main cause of morbidity and deaths in the western world. The development of atherosclerosis underlying CAD development begins early in human life. There are numerous genetic and environmental risk factors accelerating its progression which then leads to the occurrence of acute events. Despite considerable progress in determining risk factors, there is still a lot of work ahead since identified determinants are responsible only for a part of overall CAD risk. Current therapies are insufficient to successfully reduce the risk of atherosclerosis development. Therefore, there is a need for effective preventive measures of clinical manifestations of atherosclerosis since the currently available drugs cannot prevent the occurrence of even 70% of clinical events. The shift of the target from lipid metabolism has opened the door to many new therapeutic targets. Currently, the majority of known targets for anti-atherosclerotic drugs focus also on inflammation (a common mediator of many risk factors), mechanisms of innate and adaptive immunity in atherosclerosis, molecule scavengers, etc. The therapeutic potential of cyclodextrins, protein kinase inhibitors, colchicine, inhibitors of p38 mitogen-activated protein kinase (MAPK), lipid dicarbonyl scavengers, a monoclonal antibody targeting interleukin-1β, and P-selectin inhibitors is still not fully confirmed and requires confirmation in large clinical trials. The preliminary results look promising.

## 1. Introduction

Cardiovascular disease (CAD) is a serious health problem and the main cause of death and morbidity in the western world [1,2]. Atherosclerosis, which begins early in human life and is influenced by maternal risk factors, underlies CAD development [3,4]. Considerable progress has been made in explaining genetic and environmental risk factors for CAD; however, identified determinants were found to be responsible only for a part of overall risk [5]. Current therapies are insufficient to effectively reduce the risk of disease development [6]. Therefore, there is a need for effective preventive measures of clinical manifestations of atherosclerosis, including myocardial infarction, stroke, and peripheral vascular disease, since the currently available drugs cannot prevent the occurrence of even 70% of clinical events [7]. Apart from better therapeutic options, new medicaments which could limit the extent of damage resulting from acute ischemia are also highly required. The advances in next-generation sequencing, the development of platforms enabling CHD molecular characterization, the generation of big “omics”, and epigenetics data have challenged scientists to design individualized treatments and to develop new diagnostic tools which are suitable for primary and secondary prevention as well as for the treatment of patients both in early and advanced stages of this disease [5,8,9,10]. The development of precision medicine is based on the concept of “network medicine” involving the analysis of gene regulation, metabolic reactions, and protein–protein interactions (PPIs) [11]. Only the understanding of causes and mechanisms of the disease (here CAD) would allow the identification of risk factors and innovative drug targets, the design of tailored treatments, the prediction of clinical outcomes, and the establishment of preventive strategies [5]. Current therapeutic interventions are aimed at slowing down the progression of atherosclerosis and concentrate mostly on decreasing cholesterol levels [7]. However, such treatment turned out not to be effective for all patients [12]. Moreover, the results of studies indicated that inflammation may play a key role in the development of this disease [7,13]. Apart from lipid metabolism disorder and inflammatory cytokine-mediated vascular smooth muscle cell (VSMC) injury, other pathological processes (such as oxidative stress, blood coagulation in the vascular wall, fibrinolysis and renin-angiotensin) may also be involved in the development of atherosclerosis [12,14]. Since hypercholesterolemia is a crucial pathogenic factor participating in both the initiation and progression of atherosclerosis, several immunotherapeutic approaches and strategies ameliorating lipoprotein metabolism and vascular cell function have been developed [15,16,17]. In addition, some cytokines have been found to modulate the progression of atherosclerosis and, therefore, they were identified as potential therapeutic targets [18,19]. Moreover, various antigens, such as bacterial and viral antigens, as well as self-antigens, e.g., heat shock protein 60 (HSP60), have been suggested to be involved in the initiation of immune responses that stimulate atherosclerosis [15,20]. This review will focus on emerging therapies and novel use of old drugs in the treatment of cardiovascular diseases.

## 2. Cyclodextrins

**Figure 1:**
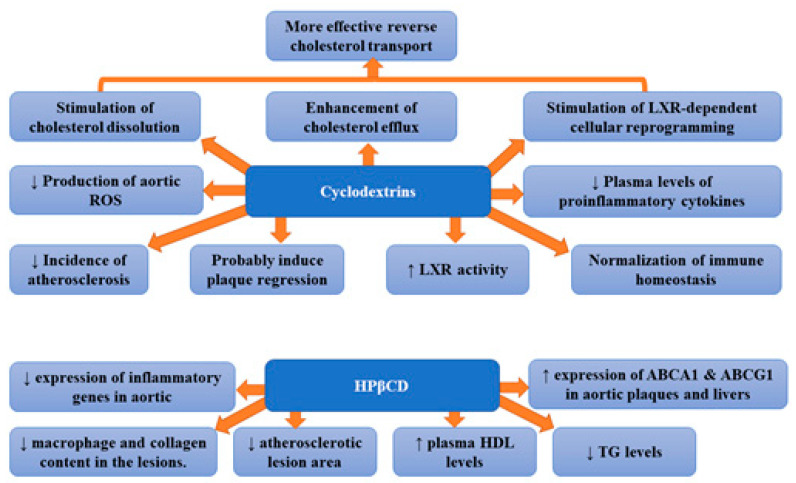
Beneficial activities of cyclodextrins and 2-hydroxypropyl-β-cyclodextrin (HPβCD) in atherosclerosis. Abbreviation: ABCA1—ATP-binding cassette transporters A1; ABCG1—ATP-binding cassette transporters G1; HPβCD-2-hydroxypropyl-β-cyclodextrin; LXR—liver X receptor; ROS—reactive oxygen species; TG—triglycerides.

Traditional treatment of atherosclerosis is focused on the reduction in LDL cholesterol [21]. The treatment of hyperlipidaemia with the use of lipid-lowering agents has been suggested to slow the progression of atherosclerosis and reduce the incidence of major coronary events [22]. Statins are the most frequently used lipid-lowering drugs; however, sometimes, their administration is associated with adverse effects. Therefore, there is a constant search for new medicines. Thus, the search for alternative methods of reducing cholesterol is in full blossom. Atherosclerosis is characterized by the remodelling of the arterial wall resulting from the accumulation of different types of lipids within the subendothelial layer. Lipid deposition as well as the presence of cholesterol crystals (formed as a result of excessive cholesterol deposition in atherosclerotic lesions) triggers the complex inflammation in the blood vessel walls [23]. Therefore, it seems that improvement in the solubility of cholesterol by CD may prove helpful in the treatment of atherosclerosis. Cholesterol crystals also stimulate the activation of the complement system and innate immune pathways as well as the formation of a neutrophil network. Therefore, it has been suggested that therapies preventing cholesterol phase transition or removing cholesterol crystals can hinder tissue inflammation and prevent disease development [24]. Cyclodextrins (CDs) can stimulate cholesterol dissolution and enhance cholesterol efflux, as well as promote liver X receptor (LXR)-dependent cellular reprogramming. Therefore, a CD-based drug delivery system has been studied in relation to atherosclerosis [12]. They are cyclic oligosaccharides made up of a variable number of glucose molecules linked by α-1,4 bonds. Based on the number of D (+)-glucose units (6, 7 or 8), CDs were divided into three groups: α-, β-, and γ-CDs. Due to the fact that CDs are considered safe and some of them have an effective and ideal molecular size, they are used as host molecules for many water-soluble molecules (especially β-CD) [25]. Cyclodextrins were found to form soluble inclusion complexes with cholesterol, increasing its solubility in aqueous solutions ~150,000-fold [6]. The foam cell macrophages containing lipoprotein-derived free cholesterol (FC) and cholesteryl ester (CE) are of key importance in the development of atherosclerotic plaque [26]. Excess cholesterol accumulated in macrophages can be released into an aqueous medium following either its conversion to more polar metabolites or to a cholesterol acceptor, e.g., HDL or apolipoprotein A-I (apoA-I) [27,28]. Several authors suggested that cyclodextrins can interact directly with membrane-embedded cholesterol since they can diffuse into the vicinity of the plasma membrane [29,30,31]. At that time, cholesterol particles can enter the hydrophobic pocket of the cyclodextrin. This process involves the formation of cyclodextrins dimers, their attachment on the interface, adsorption of cholesterol, the tilting of cyclodextrins, and, finally, the desorption of formed complexes (cyclodextrins or cholesterol) from the interface [32]. Thus, cyclodextrin can reduce cholesterol accumulation. The results of studies have confirmed that CD indeed stimulates cholesterol dissolution, boosts cholesterol efflux, as well as ameliorates cholesterol metabolism and LXR-dependent cellular reprogramming, leading to more effective reverse cholesterol transport (RCT) [12]. Cyclodextrins were demonstrated to reprogram the cells in plaques, which resulted in enhanced transport of the dissolved cholesterol away from the plaques, and decreased harmful inflammation [6]. Subcutaneous injection of CD was found to markedly decrease the incidence of atherosclerosis and even to induce the regression of established processes in mouse models [6]. Zimmer et al. [6] showed that CD treatment of murine atherosclerosis not only diminished atherosclerotic plaque size and cholesterol crystals load but was also associated with plaque regression even with a continued cholesterol-rich diet. However, CD did not change the relative plaque composition (i.e., cellularity and macrophage content). The observed effects were associated with enhanced oxysterol production in both macrophages and human atherosclerotic plaques and enhanced LXR-mediated transcriptional reprogramming which lead to improved cholesterol efflux and anti-inflammatory effects. Moreover, CD treatment decreased the production of aortic reactive oxygen species and plasma concentrations of proinflammatory cytokines which also imply CD-related reduction in the inflammatory response during atherogenesis. The authors concluded that CD treatment, which is safe in humans, can be used clinically to prevent or treat human atherosclerosis [6]. 2-hydroxypropyl-β-cyclodextrin (CD), which is a CD-derivative, was approved by the U.S. Food and Drug Administration (FDA). Its applications involve the solubilization and entrapment of numerous lipophilic pharmaceutical agents for therapeutic delivery in humans [33,34]. This molecule has proved to be beneficial in vitro; however, it remains unknown whether CDs can exert antiatherogenic effects in vivo [6]. Wang et al. [35] demonstrated that (2-hydroxypropyl)-β-cyclodextrin (HPβCD) therapy decreased levels of plasma triglyceride and inflammatory cytokine as well as increased plasma high-density lipoprotein cholesterol concentration in rabbits. Moreover, treatment with HPβCD markedly reduced the atherosclerotic lesion area and diminished macrophage and collagen content in the lesions. Furthermore, the expression levels of inflammatory genes in aortic plaques were shown to be considerably reduced, while the expression of ATP-binding cassette (ABC) transporters A1 (ABCA1) and G1 (ABCG1) in aortic plaques and livers raised significantly [35]. It appears that CDs have the ability to normalize cholesterol and immune homeostasis in the vascular system [12]. Atger et al. [36] suggested that, in human atherosclerotic lesions, the release of 7-ketocholesterol, which is a key oxidized sterol is considerably impaired. Hydroxypropyl-beta-CD was demonstrated to promote efflux of 7-ketocholesterol selectively at a rate that was 50 times higher than that of ApoA-I. The observed efflux was associated with the reduction in intracellular free and esterified 7-keto-cholesterol. These data underline the importance of extracellular sterol lysis in the efflux of cellular oxysterols as well as the mobilization of intracellular free and esterified oxysterols in macrophages loaded with ox-LDL [36]. The results of the aforementioned studies suggest that CD mediates atheroprotection by enhancing the formation of oxysterols and LXR-dependent cellular reprogramming and provide preclinical evidence that CD could be developed into an efficient atherosclerosis therapy in humans [6]. Figure 1 presents beneficial activities of cyclodextrins and 2-hydroxypropyl-β-cyclodextrin (HPβCD) in atherosclerosis.

## 3. 2-HOBA

**Figure 2:**
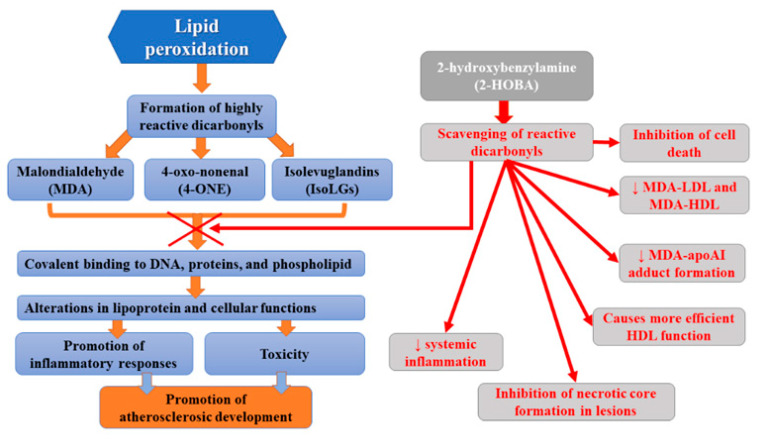
The summary of beneficial activities of 2-HOBA in atherosclerosis.

One of the recent strategies aiming to reduce atherosclerosis is the use of small molecule scavengers which selectively react with lipid dicarbonyl species [37]. These molecules prevent the modification of cellular macromolecules by reactive lipid dicarbonyls but without altering ROS levels and, therefore, without altering normal ROS signaling and function. One of such molecules, 2-hydroxybenzylamine (2-HOBA), was found to react hastily with 4-oxo-nonenal (4-ONE), malondialdehyde (MDA), and isolevuglandins (IsoLGs), but not with lipid monocarbonyls [38,39,40]. Many studies have indicated that 2-HOBA protects against oxidative stress in various disease states, including hypertension [41]. Reactive lipid dicarbonyls were demonstrated to be involved in atherogenesis, and, therefore, the antiatherogenic potential of 2-HOBA has been assessed. Tao et al. [37] observed that, in hypercholesterolemic Ldlr^−/−^ mice, the administration of 2-HOBA considerably attenuated atherosclerosis development. This molecule not only inhibited cell death but also necrotic core formation in lesions, which was associated with the formation of more stable plaques with higher collagen content and fibrous cap thickness. Moreover, they found reduced content of MDA and IsoLG adducts in the atherosclerotic lesion in 2-HOBA treated vs control mice, which was related to the scavenging of reactive dicarbonyls. Furthermore, MDA-LDL, MDA-HDL, and MDA-apoAI adduct formation was diminished. Limiting MDA modification of plasma LDL by 2-HOBA treatment is beneficial since it has been shown that such alterations of LDL molecules promote their uptake via scavenger receptors and consequent foam cell formation and inflammatory response [42,43]. 2-HOBA was found to decrease IsoLG-mediated HDL modification and dysfunction [44]. The administration of this scavenger ameliorated HDL function by reducing macrophage cholesterol stores [37]. Furthermore, 2-HOBA lowered systemic inflammation via the neutralization of reactive dicarbonyls. The limitation of atherosclerosis development following the administration of 2-hydroxybenzylamine was probably due to the scavenging of bioactive dicarbonyls since plasma cholesterol levels were found to remain unaltered. The atheroprotective mechanisms of 2-HOBA may involve the inhibition of the formation of dicarbonyl adducts of HDL proteins, resulting in preserved HDL net cholesterol efflux function. Moreover, dicarbonyl scavenging in the arterial intima resulted in the reduction in oxidative stress-induced inflammation, oxidative stress-induced apoptosis (in both endothelial cells and macrophages), and the destabilization of the plaque. The diminished cell death is probably the result of a considerably decreased inflammatory response to oxidative stress since marked reductions in serum inflammatory cytokines, such as IL-1β, were observed. Finally, 2-HOBA does not inhibit COX in mice since levels of urinary prostaglandin metabolites of prostacyclin, thromboxane, PGE2, and PGD2 remained unchanged [37]. This study demonstrated multiple antiatherogenic therapeutic effects translating into significantly reduced atherosclerosis development related to the use of 2-HOBA [37]. According to studies, HDL from patients with severe familial hypercholesterolemia (FH) also contained elevated levels of MDA adducts vs control subjects, while its activity related to decreasing macrophage cholesterol stores was impaired. Thus, it appears that 2-HOBA may prove beneficial in the treatment of atherosclerotic CVD in humans. Due to the fact that atheroprotective properties of 2-HOBA are independent of serum cholesterol levels alterations, such treatment may decrease the residual CAD risk present in patients treated with HMG-CoA reductase inhibitors [37]. Currently, this molecule, developed for use as a nutritional supplement to help maintain good health, occurs after the initial phase I studies that have reported its safety in humans [45]. It is well-tolerated at doses up to 825 mg in healthy volunteers. Adverse events reported in this trial were mild and considered unlikely to be related to 2-HOBA [45]. Furthermore, another clinical trial did not report any adverse events related to 2-HOBA [46]. Moreover, 2-HOBA acetate administration was not associated with any clinically significant findings in vital signs, ECG recordings, or clinical laboratory parameters. Preeceding in vitro safety pharmacology experiments on 2-HOBA did not indicate any compound safety concerns related to toxicity, mutagenicity, CYP induction, QT prolongation, plasma protein binding, or RBC distribution [47]. Figure 2 presents the summary of beneficial activities of 2-HOBA.

## 4. Inclisiran

Inclisiran is a chemically modified small interfering ribonucleic acid (siRNA) which targets hepatic proprotein convertase subtilisin–kexin type 9 (PCSK-9) synthesis. It is conjugated to triantennary N-acetylgalactosamine carbohydrates (GalNAc) which display a high binding affinity for liver-expressed asialoglycoprotein receptors, leading to the efficient and targeted uptake of inclisiran by hepatocytes [48]. The mechanism of action is based on RNA interference, a process during which double-stranded RNA silences the PCSK9 gene by triggering the complementary mRNA degradation and thus preventing PCSK9 protein production. The inhibition of hepatic synthesis of PCSK-9 is associated with the upregulation of many LDL receptors on the hepatocytes, resulting in the reduction in plasma LDL-C concentration [48,49,50]. Inclisiran markedly reduces LDL-C levels with an acceptable side-effect profile and an infrequent dosing regimen; it is administered subcutaneously with an initial dose, repeated at 3 months and then every 6 months [51]. The first clinical study of inclisiran (enrolling healthy volunteers who were not receiving lipid-lowering treatment) demonstrated that the maximum dose of inclisiran, 0.4 mg/kg, resulted in a mean 70% reduction in circulating PCSK9 plasma protein from baseline, compared to the placebo, and in a mean 40% decrease in LDL cholesterol levels from baseline [52]. In turn, in a ORION-1 trial, the administration of a single dose of placebo or 200, 300, or 500 mg of inclisiran on day 1, or two doses of placebo or 100, 200, or 300 mg of inclisiran on day 1 and day 90 (in combination with a maximum possible dose of a statin), resulted in a 52.6% decrease in LDL-C (two-dose 300 mg regimen) and PCSK9 levels among patients at high cardiovascular risk who had elevated LDL cholesterol levels. This reduction was similar to that achieved with PCSK-9 antibodies [53]. Another clinical trial of inclisiran included patients with atherosclerotic cardiovascular disease (ORION-10 trial) and patients with atherosclerotic cardiovascular disease or an atherosclerotic cardiovascular disease risk equivalent (ORION-11 trial) who had elevated LDL cholesterol levels despite receiving statin therapy at the maximum tolerated dose [49]. These patients were randomly assigned to receive either inclisiran (284 mg) or a placebo. Such regimen of subcutaneous inclisiran injections on day 1, day 90, and then every 6 months resulted in decreased LDL cholesterol levels by 49.9% to 52.2% at month 17. According to studies, if no more injections are given, the LDL cholesterol-lowering effects of inclisiran will be reversed at the rate of approximately 2% per month [53,54]. No clinically significant alterations in any laboratory indices or the concentrations of the cytokines (e.g., interferons α and γ, IL 6, IL 12, TNF α, and granulocyte colony-stimulating factor (G-CSF)) were observed [52]. Marked reduction in LDL-C levels following the injections of inclisiran were also observed in patients with heterozygous familial hypercholesterolemia who had been treated with a maximally tolerated dose of statin therapy, in a double-blind, randomized, placebo-controlled, phase 3 trial (ORION-9) [55]. The results of a meta-analysis evaluating data from three randomized clinical trials (3660 patients) revealed that this molecule not only reduced LDL-C levels by 51% (*p* < 0.001), total cholesterol by 37%, ApoB by 41%, and non-HDL-C by 45% in comparison to the placebo, but also lowered the incidence of major adverse cardiovascular events by 24% [56]. Considering all the obtained effects and the evidence indicating that monoclonal antibodies that target PCSK9 (evolocumab, alirocumab, and bococizumab) also reduce the incidence of major adverse cardiovascular events when used as an add-on therapy to statins, it seems plausible that inclisiran may contribute to the lowering of the incidence of cardiovascular disease in high-risk patients [57]. However, this beneficial effect must be proven in clinical trials, since it is not known whether inclisiran-related reduction in LDL-C levels will translate into diminished cardiovascular risk. Ongoing clinical trial ORION-4, conducted at approximately 180 clinical sites in the UK and the USA, is aiming to enroll 15,000 participants aged 55 years or older with pre-existing atherosclerotic cardiovascular disease who will be randomized into 300 mg of inclisiran sodium or matching placebo to assess the efficacy and safety of inclisiran ORION-4 and the effects of inclisiran on major adverse cardiovascular events (coronary heart disease (CHD) death, myocardial infarction, fatal or non-fatal ischemic stroke, and urgent coronary revascularization procedure). The results of this trial are still pending.

## 5. Molecules Targeting ANGPTL3

**Figure 3:**
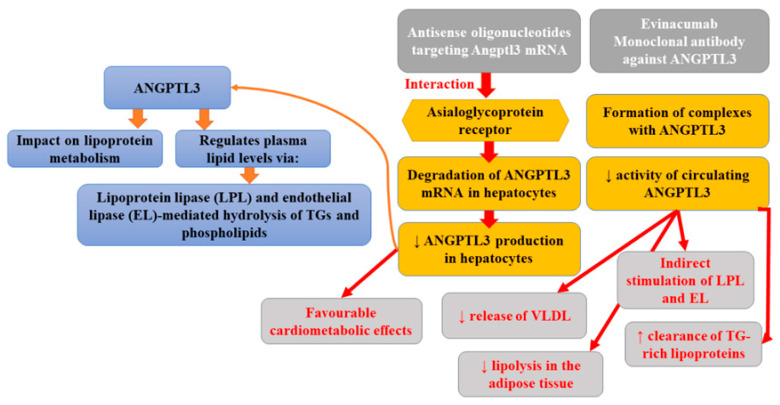
Beneficial activities of monoclonal antibody against P-selectin in atherosclerosis. Abbreviation: ANGPTL3—angiopoietin-like proteins; TG—triglycerides; VLDL—very-low-density lipoproteins.

Angiopoietin-like proteins (ANGPTLs) is a family of secreted glycoproteins that share common domain characteristics with angiopoietins (key regulators of angiogenesis). ANGPTLs are not able to bind the angiopoietin receptors expressed on endothelial cells; however, it was found that two members of this family members (ANGPTL3 and ANGPTL4) could influence lipoprotein metabolism in mice and humans and, therefore, they might have clinical importance. According to studies, ANGPTL3 regulates plasma lipid levels via a mechanism involving lipoprotein lipase and endothelial lipase-mediated hydrolysis of triglycerides (TGs) and phospholipids [22]. The inhibition of ANGPTL3 is associated with triglyceride reduction (partly mediated by the hydrolysis of triglyceride-rich lipoproteins), causing a decrease in LDL and HDL cholesterol levels [58]. Whole-exome sequencing performed as a part of the DiscovEHR study revealed that persons heterozygous for ANGPTL3 loss-of-function (LoF) variants had approximately 50% lower ANGPTL3 levels compared to non-carriers as well as 39% lower odds of CAD [59]. A complete absence of ANGPTL3 protein is associated with the presence of familial hypobetalipoproteinemia, characterized by a decrease in the levels of all lipoproteins (70% lower plasma LDL cholesterol and triglycerides) except lipoprotein(a) [60]. Moreover, it was observed that carriers of null variants of ANGPTL3 showed enhanced insulin sensitivity without a higher incidence of fatty liver disease or an apparently elevated risk of cardiovascular disease. Individuals with total ANGPTL3 deficiency were also demonstrated to have no coronary atherosclerotic plaques [61]. Plasma ANGPTL3 levels were related to the risk of myocardial infarction [62]. Antisense oligonucleotides targeting Angptl3 mRNA (ANGPTL3 ASO) interact with the asialoglycoprotein receptor (ASGR), leading to the degradation of ANGPTL3 mRNA in hepatocytes and, thus, diminished ANGPTL3 production in hepatocytes [62]. Graham et al. [63] reported that the inhibition of ANGPTL3 mRNA was associated with favorable cardiometabolic effects, not only in mouse models but also in healthy human volunteers. Pharmacologic antagonism of ANGPTL3 with a human monoclonal antibody was found to greatly diminish plasma lipid levels and atherosclerosis, which is comparable to that reported previously in the same mouse model treated with atorvastatin [64]. The preclinical studies indicated that suppression of hepatic Angptl3 protein production in mice resulted in reduced liver triglyceride content, enhanced insulin sensitivity, and limited atherosclerosis progression [63]. Evinacumab is one of the fully-humanized monoclonal antibodies IgG4 against ANGPTL3. This drug developed by Regeneron Pharmaceuticals can be administered subcutaneously or intravenously [65]. Its preparation (Evkeeza^®^) was registered in 2021 by the US Food and Drug Administration (FDA) for the treatment of homozygous familial hypercholesterolaemia (HoHF). The mechanism of this antibody involves the lowering of circulating ANGPTL3 activity, since it forms a complex with its molecule [66]. It was found that evinacumab indirectly enhanced the activity of lipoprotein lipase (LPL) and endothelial lipase (EL), boosted the clearance of triglyceride-rich lipoproteins, decreased the release of very low-density lipoproteins (VLDL), and limited lipolysis in the adipose tissue [67]. The randomized, double-blind, placebo-controlled trial assessing the safety, activity profile, and pharmacokinetics of evinacumab revealed a reduction in plasma triglyceride, LDL, and HDL levels, by 76% (day 4), 23.2% (day 15), and 18.4% (day 15), respectively [59]. Finally, Pouwer et al. [68] reported that the combination of alirocumab with evinacumab and atorvastatin not only fully inhibited the further progression of pre-existent atherosclerosis but also decreased lesion size in the aortic arch and the aortic root beyond the treatment baseline level. Moreover, such therapy improved lesion morphology and composition in APOE*3-Leiden CETP mice with pre-existent atherosclerosis. This study, for the first time, demonstrated that the use of hypolipidemic drugs combination may lead to the regression of atherosclerosis, alter macrophage content, and decrease total lesion size. Evinacumab was generally well tolerated [63]. Figure 3 presents the beneficial activities of monoclonal antibody against P-selectin in atherosclerosis.

## 6. Colchicine

To better understand and treat diverse CV disorders, we have to move beyond LDL-C alone.

Colchicine is a widely available natural product and low-cost drug displaying various anti-inflammatory properties [15]. For years, it has been used for the treatment of inflammatory diseases; however, recently, its beneficial effects in CAD have been suggested [69]. It was proposed to be a suitable treatment method for the prevention of atherosclerosis since it can block the NLRP3 inflammasome responsible for the production of interleukins IL-1b and IL-18.

**Figure 4:**
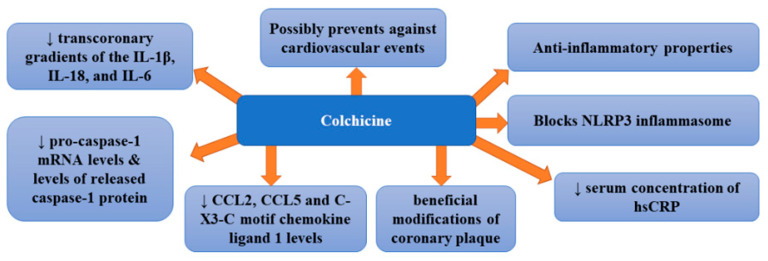
Beneficial activities of colchicine in atherosclerosis. Abbreviation: CCL2—chemokine ligand 2; CCL5—chemokine ligand 5; hsCRP—high sensitivity C-reactive protein; IL-1β,-6, -18—interleukin-1β, -6, -18; NLRP3—NOD-like receptor protein 3.

A pilot randomized controlled trial assessing the effect of colchicine (1 mg per day) compared with placebo on high sensitivity C-reactive protein in patients with acute coronary syndrome or acute stroke revealed that it did not significantly reduce absolute hs-CRP at 30 days [median 1.0 mg/L (range 0.2, 162.0) versus 1.5 mg/L (0.2, 19.8), *p* = 0.22] [70]. Moreover, no difference in platelet function was noticed. Therefore, the authors stated that colchicine did not suppress inflammation in patients with acute coronary syndrome or acute ischemic stroke. However, in a double-blind, randomized, placebo-controlled, crossover-within-subject clinical trial enrolling patients with CAD treated with low-dose colchicine (0.5 mg/day), serum concentration of hs-CRP was significantly decreased after the administration of colchicine compared with the placebo [71]. Such therapy failed to improve endothelial function in patients with CAD. Other studies provided more encouraging results. A clinical trial enrolling patients with stable coronary disease treated with 0.5 mg/day of colchicine revealed that it was effective for the prevention of cardiovascular events in patients with stable coronary disease [72]. In this study, colchicine added to statins proved to be a promising therapeutic option for patients with coronary disease. The study in which patients with ACS and stable coronary artery disease were randomized either to oral colchicine treatment (1 mg followed by 0.5 mg 1 h later) or no colchicine, 6 to 24 h before cardiac catheterization demonstrated that the administration of colchicine markedly decreased transcoronary gradients of the IL-1β, IL-18, and IL-6 cytokines in ACS patients by 40% to 88% (*p* = 0.028, 0.032, and 0.032, respectively) [73]. Therefore, it appears that colchicine can quickly and considerably lower levels of these cytokines which means that it could potentially pose a treatment option for enhanced local cardiac production of inflammatory cytokines in these patients [73]. Robertson et al. [74], who examined inflammasome activation in circulating monocytes in acute coronary syndrome (ACS) patients as well as the impact of short-term oral colchicine, found that untreated ACS patients secreted markedly higher levels of IL-18 compared with healthy controls independent of ATP stimulation (*p* < 0.05). The administration of colchicine not only significantly decreased intracellular and secreted levels of IL-1β compared with pre-treatment levels (*p* < 0.05 for both) but also reduced pro-caspase-1 mRNA levels by 57.7% and released caspase-1 protein levels by 30.2% compared with untreated patients (*p* < 0.05 for both). A recent study of patients with the acute coronary syndrome randomly assigned to colchicine (1.5 mg p.o.) [75] showed that colchicine treatment was associated with significantly lower transcoronary levels of chemokine ligand 2 (CCL2), CCL5, and C-X3-C motif chemokine ligand 1 (CX3CL1) (*p* < 0.05). Colchicine was also found to inhibit CCL2 gene expression in stimulated monocytes in vitro, while in vivo it diminished intracellular levels of all three chemokines (*p* < 0.01) and impaired monocyte chemotaxis (*p* < 0.05). Since colchicine therapy decreases the local production of coronary chemokines, it seems to be beneficial for patients with ACS. In turn, the purpose of another study was to assess whether low-dose colchicine exerted any impact on the plaque modification [76]. In this prospective non-randomized observational study, patients with recent ACS (<1 month) received either 0.5 mg/day colchicine plus optimal medical therapy or optimal medical therapy alone. Vaidya et al. [76] found that colchicine treatment markedly diminished low-attenuation plaque volume (LAPV) (mean 15.9 mm^3^ [−40.9%] vs. 6.6 mm^3^ [−17.0%]; *p* = 0.008), which is a marker of plaque instability and strong predictor of adverse cardiovascular events. This therapy was also associated with reduced hsCRP levels (mean 1.10 mg/L [−37.3%] vs. 0.38 mg/L [−14.6%]; *p* < 0.001) compared with controls. However, no differences in total atheroma volume or low-density lipoprotein levels were observed between groups. This study also indicated linear association (*p* < 0.001) and robust positive correlation (r = 0.578) between change in LAPV and hsCRP. Vaidya et al. [76] provided the first evidence of beneficial modifications of coronary plaque by colchicine, which was independent of high-dose statin intensification therapy and low-density lipoprotein reduction. Observed favorable changes in plaque morphology were suggested to be related to the anti-inflammatory properties of colchicine. Therefore, colchicine may prove advantageous in secondary prevention in post-ACS patients; however, this thesis requires further confirmation. The therapy with colchicine was also found to reduce the risk of cardiovascular events, such as angina, myocardial infarction, and death [77]. Recently, two large clinical trials: the Colchicine Cardiovascular Outcomes Trial (COLCOT) and LoDoCo2 have been conducted. They included >10,000 patients and demonstrated decreased cardiovascular risk, both in patients after myocardial infarction and in those with chronic stable coronary disease following the administration of colchicine [77,78]. Such beneficial effects have been confirmed by a recent meta-analysis which indicated a 38% reduction in the risk of myocardial infarction, a 62% decrease in the risk of stroke and a 44% lowering of risk of urgent coronary revascularization [79]. The greatest effects were obtained when colchicine treatment was initiated within the first three days after myocardial infarction (48% in the risk of the composite primary endpoint in COLCOT) and resulted from the decreased occurrence of myocardial infarction, stroke, and urgent hospitalization for angina, leading to coronary revascularization [80]. Figure 4 presents beneficial activities of colchicine in atherosclerosis.

## 7. Canakinumab

Canakinumab, a fully human monoclonal antibody targeting interleukin-1β, is a cytokine that is vital for the inflammatory response launching the interleukin-6 signaling pathway [7]. Many previous studies performed on animals and in vitro confirmed that the proinflammatory cytokine interleukin-1β plays various roles in the development of atherothrombotic plaque. Apart from inducing procoagulant activity, it also stimulates monocyte and leukocyte adhesion to vascular endothelial cells, as well as the growth of vascular smooth-muscle cells [81,82]. Kirii et al. [83] demonstrated that the deficiency of interleukin-1 β reduced the severity of atherosclerosis in ApoE-deficient mice. While, in pigs fed with cholesterol, the exposure to exogenous interleukin-1β was associated with intimal medial thickening [84]. Interleukin-1β is activated by NOD-like receptor protein 3 (NLRP3) inflammasome in a process that is stimulated by cholesterol crystals, neutrophil extracellular traps, arterial flow patterns, as well as tissue hypoxia [85,86,87]. Mendelian randomization studies suggested that the activation of interleukin-1β and subsequent stimulation of downstream interleukin-6-receptor signaling pathway was a casual mechanism for atherothrombosis [88,89]. In turn, studies of clonal hematopoiesis revealed that interleukin-1β is involved in a process by which bone marrow activation accelerates atherosclerosis [90,91].

Canakinumab was found to exert anti-inflammatory effects. In a phase 2 trial involving patients with diabetes who were at high vascular risk, the administration of canakinumab significantly decreased levels of high-sensitivity C-reactive protein and interleukin-6 as compared with placebo but had no impact on lipid levels [92]. Despite the lack of significant reduction in cholesterol levels, the observed effect of the administration of canakinumab for 3 months was comparable to that related to the use of monoclonal antibodies targeting proprotein convertase subtilisin–kexin type 9 (PCSK9) [93,94]. Similar effects were also found in the Canakinumab Anti-inflammatory Thrombosis Outcome Study (CANTOS), a randomized, double-blind, placebo-controlled trial enrolling 10,061 stable patients with a history of myocardial infarction and a high-sensitivity C-reactive protein level of 2 mg/L or more followed-up for 3.7 years (at a median) [7]. Before the treatment with canakinumab, each tertile increase in IL-6 before canakinumab treatment was associated with a 50% increase in the risk of MACE (95% CI 34–67%, *p* < 0.0001). Similar effects were observed for CV death, all-cause mortality, and the combined endpoint of all vascular events (revascularization procedures and hospitalization for congestive heart failure). The median decrease from baseline in the high-sensitivity C-reactive protein level was 26% points greater in the group receiving the 50-mg dose of canakinumab, 37% points higher in the 150-mg group, and 41% points greater in the 300-mg group compared with the placebo group. The administration of a 50-mg dose of canakinumab did not affect the occurrence of primary cardiovascular endpoint compared with placebo; however, it lowered the risk of the key secondary cardiovascular endpoint (by 17%) (4.29 vs. 5.13 events per 100 person-years). Canakinumab at a dose of 150 mg (once every 3 months) resulted in a markedly diminished rate of recurrent cardiovascular events compared to placebo, independent of lipid-level lowering [7]. Based on study results, Ridker et al. [7] concluded that direct inhibition of NLRP3 function or therapies altering downstream interleukin-6 signaling may prove beneficial in reducing cardiovascular risk.

However, their study also revealed that, in patients treated with canakinumab, the incidence of fatal infection and sepsis and a decrease in platelet counts with no increase in bleeding risk were significantly increased. In the CANTOS study, the risk factors for infections in canakinumab-treated patients included age and chronic kidney disease [95]. The higher risk of infections in patients treated with canakinumab was suggested to be decreased by intensifying the awareness of infections during such therapy as well as the initiation of early antimicrobial therapy for suspected bacterial infections. The increased incidence of sepsis and fatal infections in patients treated with canakinumab may be partly explained by the fact that the development of sepsis is associated with increased levels of many pro-inflammatory cytokines, including i.a. IL-7, IL-8, IL-10, IL-13, IFN-γ, MCP-1, and TNF-α [96]. Moreover, according to some studies, IL-1β is protective in many bacterial infections. IL-1β inhibition (e.g., with the use of receptor antagonists) was found to reduce the clearance of invading bacteria [97,98]. Such a mechanism may explain the higher incidence of sepsis in patients treated with antibody targeting interleukin-1β. No difference in all-cause mortality was observed between the canakinumab groups and the placebo group [7].

**Figure 5:**
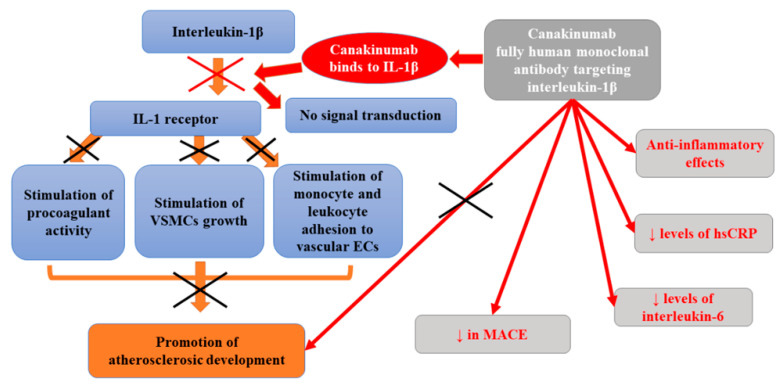
Beneficial activities of canakinumab in atherosclerosis. Abbreviations: ECs—endothelial cells; hsCRP—high sensitivity C-reactive protein; MACE—major adverse cardiovascular events; VSCM—vascular smooth muscle cell.

At the same time, patients in this study had considerably reduced cancer mortality compared to a placebo group. Such finding is in agreement with experimental data pointing to the role of interleukin-1 in the progression and invasiveness of some tumors, especially lung cancer [99,100]. In general, canakinumab diminished IL-6 levels at 3 months by 34.9% (*p* < 0.001), compared with placebo, in a dose-dependent manner (24.5%, 35.8%, and 42.7% for the 50 mg, 150 mg, and 300 mg doses, respectively) [101]. Ridker et al. [101] also studied to what extent beneficial cardiovascular outcomes related to the administration of canakinumab are mediated through interleukin-6 (IL-6) signaling. They observed that a subset of 4833 of stable atherosclerotic patients participating in CANTOS receiving canakinumab who achieved on-treatment IL-6 levels below the study median value of 1.65 ng/L experienced a 32% reduction in major adverse cardiovascular events [MACE, multivariable-adjusted hazard ratio (HR_adj_) 0.68, 95% confidence interval (CI) 0.56–0.82; *p* < 0.0001], a 30% decrease in MACE plus the additional endpoint of hospitalization for unstable angina requiring urgent revascularization (MACE+, HR_adj_ 0.70, 95% CI 0.59–0.84; *p* < 0.0001), a 52% lowering in cardiovascular mortality (HR_adj_ 0.48, 95% CI 0.34–0.68; *p* < 0.0001), and a 48% reduction in all-cause mortality (HR_adj_ 0.52, 95% CI 0.40–0.68; *p* < 0.0001) with prolonged treatment compared to placebo group. However, those with on-treatment IL-6 levels ≥1.65 ng/L after the first dose of canakinumab did not significantly benefit from any of these endpoints. In turn, Russell et al. [102] analyzed the impact of canakinumab (150 mg subcutaneously, up to 12 months) on the extensive atherosclerotic plaque in patients with symptomatic peripheral artery disease (PAD). Their randomized, placebo-controlled trial demonstrated that the concentration of markers of systemic inflammation (interleukin-6 and high-sensitivity C-reactive protein) decreased as early as 1 month after treatment. However, MRI failed to show any differences in plaque progression in the superficial femoral artery between placebo-treated or canakinumab-treated patients. The inhibition of interleukin-1β is only one of the therapeutic options as many other potential inflammatory pathways could serve as targets for atheroprotection [94]. Figure 5 presents beneficial activities of canakinumab in atherosclerosis.

## 8. Ziltivekimab

The aforementioned clinical trial (CANTOS) have indicated that the IL-6 signaling pathway decreased cardiovascular event rates independent of LDL lowering [101]. In this trial, the observed clinical benefit was directly associated with the extent of downstream IL-6 reduction, which implies that IL-6 may be the primary target for atheroprotection [103]. The role of IL-6 signaling in the development of various forms of atherosclerosis (MI, PAD, and aortic aneurysm formation) was confirmed by GWAS and PHEWAS data. The safety and efficacy of IL-6 inhibition among individuals at high atherosclerotic risk but without a systemic inflammatory disorder remain unknown, and therefore a randomized, double-blind, placebo-controlled phase 2 RESCUE trial focused on these issues [104]. This trial assessed the effects of ziltivekimab, a fully human monoclonal antibody directed against the IL-6 ligand with extended half-life technology, on multiple biomarkers of inflammation and thrombosis in patients at high cardiovascular risk with moderate to severe chronic kidney disease (CKD) and elevated hsCRP. Ziltivekimab has earlier completed two early-stage trials in chronic kidney disease patients in which its administration reduced the levels of C-reactive protein (CRP). Participants in the RESCUE trial were randomized (1:1:1:1) to subcutaneous administration of placebo or ziltivekimab in a dose of 7.5 mg, 15 mg, or 30 mg every 4 weeks up to 24 weeks [104]. Ziltivekimab was found to significantly decrease various biomarkers of systemic inflammation and thrombosis involved in the stimulation of the atherothrombotic process development [101]. After 12 weeks of treatment, median hsCRP levels were diminished by 77% in the 7.5-mg group, 88% in the 15-mg group, and 92% in the 30-mg group compared with 4% in the placebo group. The effects remained stable over the 24-week treatment period. Moreover, the reduction in hsCRP with the use of ziltivekimab was demonstrated to be twice as large as that observed in the CANTOS trial of canakinumab. The anti-inflammatory beneficial effects of ziltivekimab appeared not to be related to hepatic toxicity, bone marrow suppression, infectious risk, or change in atherogenic lipid levels. It had no impact on the total cholesterol to HDL cholesterol ratio. Ziltivekimab was demonstrated to be well tolerated and there were no serious injection-site reactions. Apart from hsCRP levels, dose-dependent reductions were also found for fibrinogen, serum amyloid A, haptoglobin, secretory phospholipase A2, and lipoprotein(a) (from 16.4% to 25.0% in all treatment groups) [104]. The use of ziltivekimab slightly increased apolipoprotein B (APOB), high-density lipoprotein (HDL) cholesterol, and apolipoprotein A1 (APOA1).

The success of the RESCUE trial resulted in the launching of the Ziltivekimab Cardiovascular Outcomes Study (ZEUS) a phase 3 trial that will be conducted in 6200 ASCVD patients with CKD (stage 3–4) and elevated CRP ≥ 2 mg/L [101].

Apart from improving markers of inflammation, ziltivekimab reduces ESA requirements, and rises serum albumin in patients on hemodialysis with inflammation and hyporesponsiveness to ESA therapy [105].

## 9. Losmapimod

**Figure 6:**
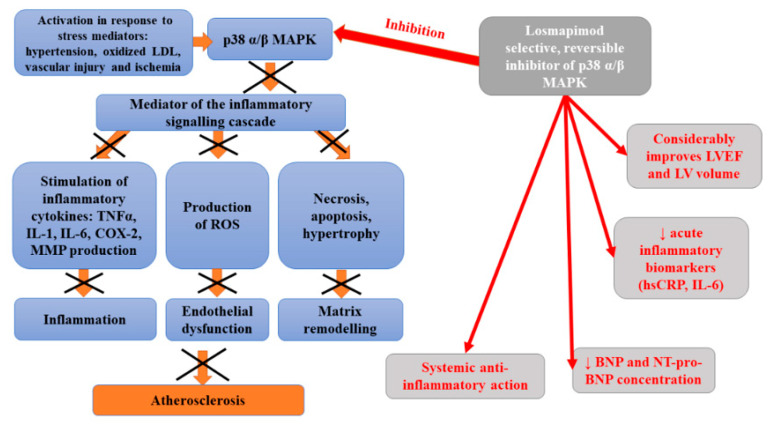
Beneficial activities of losmapimod in atherosclerosis. Abbreviation: BNP—brain natriuretic peptide; COX-2—cyclooxygenase 2; hsCRP—high sensitivity C-reactive protein; IL-1,-6—interleukin-1,-6; LVEF—left ventricular ejection fraction; LV—left ventricular; MAPK—mitogen-activated protein kinase; MMP—metalloproteinases; NT-pro-BNP—N-terminal prohormone of brain natriuretic peptide; ROS—reactive oxygen species; TNFα—tumor necrosis factor-alpha.

Losmapimod is a novel, selective, reversible, orally administered inhibitor of p38 mitogen-activated protein kinase (MAPK) α and β [106]. MAPK kinase, expressed in vascular endothelium, smooth muscle, macrophages, and cardiac myocytes, becomes activated in response to stress mediators, such as hypertension, oxidized low-density lipoprotein cholesterol, vascular injury, and ischemia [107]. An intracellular kinase is an important mediator of the inflammatory signaling cascade which is responsible for the activation of cytokine (tumor necrosis factor-alpha (TNFα), interleukin-1, IL-6) production as well as cyclooxygenase 2 and metalloproteinases during acute coronary syndrome [108,109]. Preliminary data suggested that the inhibition of p38 MAPK may disturb inflammatory processes within the vascular wall, resulting in the stabilization of atherosclerotic plaques, and diminished risk of plaque rupture. Elkhawad et al. [109] analyzed the relationship between the p38 mitogen-activated protein kinase cascade and the initiation and progression of inflammatory diseases, including atherosclerosis. In this study, a considerable decrease in average tissue-to-background ratio, as well as the reduction in inflammatory biomarkers levels and 18F-fluorodeoxyglucose uptake in visceral fat, were observed in patients who received a high dose of losmapimod compared to the placebo. Thus, it was suggested that losmapimod may exert a systemic anti-inflammatory action. In turn, Newby et al. [110] studied the impact of p38 MAPK inhibition on myocardial condition in patients with non-ST-segment elevation myocardial infarction (NSTEMI). They observed lower concentrations of high sensitivity C-reactive protein and B-type natriuretic peptide in patients treated with losmapimod, compared to a placebo group. The safety of 12-week administration of losmapimod and its impact on systemic inflammation, infarct size, and cardiac function has been assessed in phase II, randomized, double-blind, placebo-controlled study enrolling 526 subjects with non-ST-segment elevation myocardial infarction (NSTEMI) [106]. This study indicated a marked reduction in acute inflammatory biomarkers (high sensitivity C-reactive protein; hsCRP and interleukin 6; IL-6) in patients treated with losmapimod compared to placebo. This trial demonstrated a non-significant trend toward the lower occurrence of major adverse cardiovascular events (MACE) in treated subjects, associated with the reduction in myocardial infarction (MI). Moreover, such therapy considerably improved left ventricular ejection fraction (LVEF) and left ventricular (LV) volumes. However, the outcomes of Phase III LATITUDE-TIMI 60 randomized, placebo-controlled, double-blind, parallel-group trial conducted at 322 sites in 34 countries failed to indicate the reduction in the risk of major ischemic cardiovascular events in patients hospitalized with an acute MI or at least one additional predictor of cardiovascular risk [111]. Moreover, no evidence that losmapimod decreases the incidence of any secondary outcomes, including all-cause mortality, was obtained. Figure 6 presents beneficial activities of losmapimod in atherosclerosis.

## 10. MK2206

Numerous studies have suggested that Akt may play an important role in the pathogenesis and progression of atherosclerosis and cancer [112,113,114]. AKT signaling regulates the activity of sterol regulatory element-binding proteins (SREBPs) (via altering SREBPs gene transcription, protein maturation, or protein stability), which, in turn, control the expression of LDLR mainly at the level of transcription [115]. Therefore, the effectiveness of MK2206, which is a highly selective allosteric inhibitor of Akt, has been assessed in animal models. The use of an allosteric inhibitor is a new strategy that helps to decrease possible off-target toxicity. Moreover, MK2206 was found to be able to inhibit all three isoforms of Akt kinases (Akt1, Akt2, and Akt3) with specificity and acceptable toxicities [116]. It binds in a cavity formed at the interface of the catalytically active kinase domain and the regulatory pleckstrin homology domain, securing the kinase in a closed, inactive conformation [117,118]. In in vivo (ApoE^−/−^ mice) and in vitro studies, Tang et al. [112] demonstrated the protective role of MK2206 in CVD. They observed that MK2206 exerted favourable effects on the cardiovascular system, it successfully attenuated atherosclerosis and protected against vascular toxicity in vivo. Moreover, this inhibitor not only reduced the accumulation of lipids in macrophages via the stimulation of cholesterol efflux, but also limited VSMC migration and proliferation of Raw264.7, VSMC, and HUVEC cells. Therefore, it appears that MK2206 can diminish inflammatory responses in macrophages, VSMC, and endothelial cells. The results of studies have indicated that MK2206 suppressed oxLDL-induced proliferation of endothelial cells and reversed the stimulating effect of VSMC proliferation and migration mediated by miR-21 overexpression [119,120]. Indeed, Tang et al. [112] found demonstrated that MK2206 inhibited proliferation of Raw264.7, VSMC, and HUVEC cells as well as VSMC migration. However, they did not notice any significant impact of MK2206 on the necrotic core area and fibrous cap area, which determine the stability of vulnerable plaques. Their findings may suggest that studied Akt inhibitor primarily plays a role in early atherosclerosis. Furthermore, other studies demonstrated the ability of MK2206 to reverse the decrease in cholesterol efflux mediated by free fatty acid in macrophages and to abolish lipid accumulation and cholesterol efflux induced by ADPβ in VSMCs [121,122]. It was observed that MK2206 can reduce lipid deposition in macrophages via enhancing cholesterol efflux but not lipid uptake [112]. Akt2 was found to affect macrophage migration, inflammation, and lipid deposition, while its deficiency showed less atherosclerosis in Ldl receptor-Null (Ldlr^−/−^) mice [123,124]. Therefore, it seems that diminished lipid deposition and inflammation following the administration of MK2206 could be attributed to the decreased activity of Akt2. MK2206-related reduction in inflammatory response in vitro and in vivo was found to be mediated by the mRNA decay of inflammatory factors. In turn, the antimigrative and antiproliferative effects of MK2206 on VSMCs could be related to the reduction in Akt1 activity [125,126]. Animal study revealed that miR-223 could regulate the expression levels of multiple upstream factors of Akt and, thus, modulate activation of Akt, consequently leading to heart cells hypertrophy, while the use of MK2206 inhibited miR-223-induced Akt activation [127]. Therefore, it seems that cardiac-specific over-expression of miR-223 in vivo could induce physiological heart hypertrophy; however, whether other miRNA may promote pathways involved in vascular remodeling (and which can be inhibited by MK2206) remains unknown. It seems that ApoE knockout animals is a good model to check whether miRNA mediates the effect of MK2206 on vascular remodeling.

**Figure 7:**
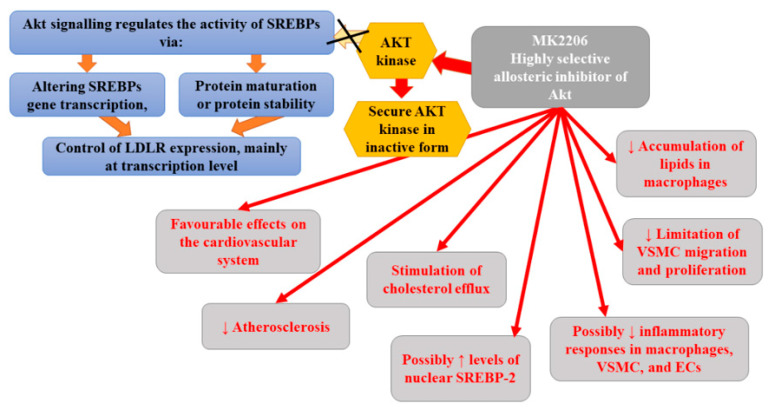
Beneficial activities of monoclonal antibody against P-selectin in atherosclerosis. Abbreviation: SREBPs—sterol regulatory element-binding proteins; ECs—endothelial cells; VSCM—vascular smooth muscle cell.

Bjune et al. [115] demonstrated that MK-2206 affected the expression of LDLR. They demonstrated that it activated SREBP-2 and stimulated the reduction in LDLR and LDL- C levels in a manner that was independent of 3-hydroxy-3-methylglutaryl-CoA reductase (HMGCR) inhibition, endoplasmic reticulum (ER) stress, or apoptosis. Moreover, MK2206 boosted the cellular uptake of LDL-C via the induction of LDLR mRNA and LDLR protein [115]. In contrast to statins, MK2206-mediated induction of LDLR was shown to be independent of intracellular cholesterol status. Based on obtained results, Bjune et al. [115] hypothesized that MK2206 circumvents the dependency on low intracellular cholesterol levels to promote the expression of LDLR via triggering a signaling cascade that stimulates the activation of SREBP-2. Therefore, it can upregulate LDLR even in the case of sterol negative feedback regulation of LDLR expression. Moreover, according to the authors, allosteric inhibition is associated with the activation of SREBP-2 rather than with the stimulation of AKT [115]. MK-2206 was suggested to increase the amount of nuclear SREBP-2 by promoting the proteolytic processing of nascent SREBP-2. All the aforementioned findings support the thesis that MK2206 may prove beneficial in the treatment of atherosclerosis. However, due to the fact that Akt activity is central for the proper functioning of many cellular processes, including insulin signaling, the prolonged use of MK2206 may be associated with increased incidence of serious adverse effects, such as hyperglycemia [128]. Indeed, clinical trials with MK2206 in cancer patients demonstrated a higher prevalence of mostly mild and transient hyperglycemia [129,130]. Clinical trials are yet to assess the effectiveness of MK2206 in the treatment of hypercholesterolemia and atherosclerosis. Figure 7 presents the beneficial activities of Akt inhibitors in atherosclerosis.

## 11. Monoclonal Antibody against P-Selectin

P-selectin is a cell adhesion molecule and a component of the membrane of Weibel–Palade bodies of endothelial cells and the α-granules of platelets. Once activated, it can be promptly translocated to the cell surface where it interacts with P-selectin glycoprotein ligand 1. P-selectin is involved in leukocyte tethering and rolling on the vessel wall and consequent extravasation as well as the stimulation of platelet rolling and adhesion to the activated vessel wall [131]. The results of studies have indicated that soluble P-selectin has prothrombotic and procoagulant properties [132,133]. Elevated plasma levels of soluble P-selectin has been observed in many cardiovascular disorders, including coronary artery disease, venous thromboembolism, diabetes, and hypertension [134,135,136,137]. The results of animal studies demonstrated that the blockade of P-selectin functions inhibited the development of atherosclerosis, fibrin deposition, thrombus growth, as well as ischemia-induced tissue injury [138,139,140,141].

Inclacumab is a recombinant human monoclonal antibody of the immunoglobulin G4 subclass directed against human P-selectin. Two single-point mutations (L235E and S228P) were introduced into the Fc part to improve structural stability, but also to prevent antibody-dependent cell-mediated cytotoxicity. It was found to have a high affinity towards human P-selectin and high selectivity compared with the other members of the selectin family (E-selectin and L-selectin) [142]. High selectivity is necessary to avoid immunocompromised phenotype (observed in the study of double-selectin knockout mice) [143]. Inclacumab, which inhibits P-selectin-mediated functions, has been reported to display anti-inflammatory and antithrombotic properties. Ex vivo studies demonstrated that it repressed the adhesion of leukocytes to endothelial cells as well as reduced leukocyte adhesion to platelet monolayers and the formation of platelet–leukocyte aggregates (PLA) [144]. Since inclacumab hampers inflammatory and thrombotic cascades, it was suggested to have the potential to treat atherosclerosis. The first evidence confirming its clinical efficacy comes from a SELECT-ACS trial which demonstrated that inclacumab reduced myocardial damage after percutaneous coronary intervention (PCI) in patients with non-ST-segment elevation myocardial infarction (non-STEMI) [145]. In this trial, 544 non-STEMI patients were divided into three groups: the first group was treated with inclacumab in a dose of 5 mg/kg, the second group was treated with inclacumab in a dose of 20 mg/kg, and the third group received placebo. Inclacumab in a dose of 20 mg/kg markedly decreased TnI and CK-MB levels, compared to the placebo. Therefore, authors stated that it seems to be a potential and promising treatment option for the reduction in myocardial damage after PCI in patients with NSTEMI [145]. Subsequent analysis within SELECT-ACS found that inclacumab appears to be more beneficial in the case that the drug infusion takes place less than 3 h before PCI [146].

**Figure 8:**
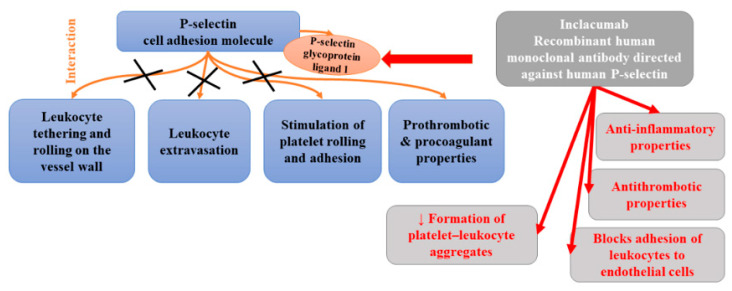
Beneficial activities of monoclonal antibody against P-selectin in atherosclerosis.

The blockade of P-selectin at the surface of platelets by inclacumab was also found to prevent the formation of platelet–leukocyte aggregates (PLA). Increased concentrations of circulating PLA, which are directly associated with platelet expression of P-selectin, have been observed in various cardiovascular diseases [147]. Schmitt et al. [142] found that inclacumab inhibited PLA formation. Both platelet–leukocyte aggregate inhibition and soluble P-selectin occupancy displayed dose-dependency and were strongly correlated to inclacumab plasma concentrations. Moreover, this antibody appeared to be safe and well-tolerated. Figure 8 presents beneficial activities of monoclonal antibody against P-selectin in atherosclerosis.
**Drug Name** **Intervention****Intervention****Type of Study****Most Relevant Effects of Drug****Safety/Potential Serious Side Effects****Ref.**2-hydroxypropyl-β-cyclodextrin
Animal study (mice)-Reduction in atherosclerotic plaque size and CC load-Plaque regression even in mice on a cholesterol-rich diet-Increased oxysterol production in macrophages and human atherosclerotic plaques-No change in the relative plaque composition-Stimulation of LXR–mediated transcriptional reprogramming to improve cholesterol efflux-Decreased the production of aortic ROS and plasma concentrations of proinflammatory cytokines—anti-inflammatory effectsGenerally well tolerated, but the latest research indicated the increased risk of ototoxicity in the form of iatrogenic hearing loss [148][6]
Animal study (rabbits fed a high-fat diet)-Reduced plasma levels of TG and inflammatory cytokines-Increased plasma HDL-C-Reduction in atherosclerotic lesion area-Diminished macrophage and collagen content in the lesions-Significant decrease in expression levels of inflammatory genes in aortic plaques-Increased expression of ABCA1 and ABCG1 in aortic plaques and liver[35]2-hydroxybenzylamine (2-HOBA)
Animal study (hypercholesterolemic Ldlr^−/−^ mice) – model of FH-Decreased atherosclerosis by 60% in en face aortas of mice compared to mice treated with vehicle or a nonreactive analogue (4-HOBA)-Reduced MDA-LDL and MDA-HDL levels-Increased capacity of HDL to reduce macrophage cholesterol-Reduction in MDA- and IsoLG-lysyl content in atherosclerotic aortas-Diminished inflammation and plaque apoptotic cells-Stimulation of efferocytosis and features of stable plaques2-HOBA acetate was safe and well-tolerated at doses up to 825 mg in healthy human volunteers [45]. Adverse events reported in this trial were mild and considered unlikely to be related to 2-HOBA[37]InclisiranOne dose (200, 300, or 500 mg on day 1) or 2 doses (100, 200, or 300 mg on days 1 and 90) of inclisiran sodium or placeboRandomized, double-blind, placebo-controlled multicentre phase 2 clinical trial (ORION-1)-Treatment with inclisiran resulted in durable reductions in LDL-C-Time to return to within 20% of change from baseline for LDL-C levels and time-averaged LDL-C reductions over 1 yearWell tolerated Mild rash in some participants [54]One intravenous dose (doses ranging from 0.015 to 0.400 mg/kg) or placeboRandomised, single-blind, placebo-controlled, phase 1 dose-escalation study in healthy adult volunteers with serum LDL cholesterol of 3.00 mmol/L or higher-A mean 70% reduction in circulating PCSK9 plasma protein (*p* < 0·0001)-A mean 40% reduction in LDL cholesterol from baseline relative to placebo (*p* < 0·0001)No drug-related serious adverse events. Mild to moderate treatment-emergent adverse events A transient mild, macular, erythematous rash[52]Subcutaneous injection. Single dose of placebo or 200, 300, or 500 mg of inclisiran or two doses (at days 1 and 90) of placebo or 100, 200, or 300 mg of inclisiranPhase 2, multicentre, double-blind, placebo-controlled, multiple-ascending-dose trial. Patients at high risk for cardiovascular disease who had elevated LDL cholesterol levels-Mean reductions in LDL cholesterol levels: 27.9 to 41.9% after a single dose of inclisiran and 35.5 to 52.6% after two doses (*p* < 0.001 for all comparisons vs. placebo)-At day 240, PCSK9 levels were 56.1% lower and LDL cholesterol levels 47.2% lower than at baseline, with a mean absolute reduction in LDL cholesterol level of 58.9 mg/dL (1.52 mmol/L)Rarely symptoms of immune activation. Rare transient elevations in hepatic enzyme levels[53]
ORION-10 trial: patients with atherosclerotic cardiovascular disease with elevated LDL cholesterol levels despite receiving statin therapy at the maximum tolerated dose-At day 510, a reduction in LDL cholesterol levels by 52.3% (95% confidence interval [CI], 48.8 to 55.7, with corresponding time-adjusted reductions of 53.8% (95% CI, 51.3 to 56.2) (*p* < 0.001 for all comparisons vs. placebo)-Decreased levels of triglycerides and lipoprotein(a) and increased HDL cholesterol levels at day 510injection-site adverse events were more frequent with inclisiran than with placebo (2.6% vs. 0.9%) Adverse reactions were generally mild, and none were severe or persistent[49]
ORION-11 trial: patients with atherosclerotic cardiovascular disease or an atherosclerotic cardiovascular disease risk equivalent with elevated LDL cholesterol levels despite receiving statin therapy at the maximum tolerated dose-At day 510, reduction in LDL cholesterol levels by 49.9% (95% CI, 46.6 to 53.1) with corresponding time-adjusted reductions of 49.2% (95% CI, 46.8 to 51.6) (*p* < 0.001 for all comparisons vs. placebo)-Decreased levels of triglycerides and lipoprotein(a) and increased HDL cholesterol levels at day 510injection-site adverse events were more frequent with inclisiran than with placebo (4.7% vs. 0.5%) Adverse reactions were generally mild, and none were severe or persistent[49]Antisense oligonucleotides targeting Angptl3 mRNASubcutaneous injections of placebo or an antisense oligonucleotide targeting ANGPTL3 mRNA in a single dose (20, 40, or 80 mg) or multiple doses (10, 20, 40, or 60 mg/week for 6 weeks)Clinical trial including 44 human participants (with TG levels of either 90 to 150 mg per deciliter [1.0 to 1.7 mmol per liter] or >150 mg per deciliter, depending on the dose group) -Dose-dependent reduction in levels of hepatic Angptl3 mRNA, Angptl3 protein, TG, and LDL cholesterol-Reduced liver TG content-Hampered atherosclerosis progression-Increased insulin sensitivity-Multiple-dose groups: reduced levels of ANGPTL3 protein (46.6 to 84.5% from baseline, *p* < 0.01 for all doses vs. placebo), TG (33.2 to 63.1%), LDL cholesterol (1.3 to 32.9%), VLDL (27.9 to 60.0%), non–HDL cholesterol (10.0 to 36.6%), apolipoprotein B (3.4 to 25.7%), and apolipoprotein C-III (18.9 to 58.8%) after 6 weeks of treatmentNo serious adverse events. Rarely dizziness or headache[63]Evinacumab (fully human anti-ANGPTL3 monoclonal antibody)Placebo subcutaneously (75, 150, or 250 mg) Intravenously (5, 10, or 20 mg/kg)A phase 1, first-in-human, randomized, placebo-controlled, double-blind, ascending single-dose clinical trial Healthy persons with a fasting TG level of 150 to 450 mg/dL (1.7 to 5.1 mmol/L) or an LDL cholesterol level of 100 mg/dL (2.6 mmol/L) or greater-Dose-proportional magnitude and duration of lipid reductions (placebo-corrected)-The maximal changes in lipid levels found among patients who received a dose of 20 mg/kg intravenously were as follows: triglycerides, −76.0% (day 4); directly measured LDL cholesterol, −23.2% (day 15); and HDL cholesterol, −18.4% (day 15).The most frequent adverse event—headache (11%). Transient, single elevations of the alanine aminotransferase level to more than 3 times the upper limit of the normal range[59]Atorvastatin Alirocumab; EvinacumabDiet alone (control) or atorvastatin; atorvastatin and alirocumab; atorvastatin and evinacumab; or atorvastatin, alirocumab, and evinacumab (triple therapy)] for 25 weeksAPOE*3-Leiden.CETP mice (model for hyperlipidemia)-All interventions reduced plasma total cholesterol (37% with atorvastatin to 80% with triple treatment; all *p* < 0.001).-Triple treatment decreased non-HDL-C to 1.0 mmol/l (91% difference from control; *p* < 0.001).-Triple treatment regressed lesion size versus baseline in the thoracic aorta by 50% and the aortic root by 36% (both *p* < 0.05 vs. baseline), decreased macrophage accumulation through reduced proliferation, and abated lesion severity.-[68]Colchicine1 mg daily for 30 daysA pilot randomized controlled trial 80 patients with ACS or acute ischemic stroke-No significant reduction in absolute hs-CRP at 30 days [median 1.0 mg/L (range 0.2, 162.0) versus 1.5 mg/L (0.2, 19.8), *p* = 0.22]-No difference in platelet functionOccurrence of diarrhoea (X(2) 4.14, *p* = 0.04)[70]Low-dose colchicine (0.5 mg/day) or a placebo for 7 daysDouble-blind, randomized, placebo-controlled, crossover-within-subject clinical trial 28 patients with CAD -Significantly decreased serum concentration of hs-CRP compared with placebo [median (interquartile range): 0.04 (0.02–0.08) mg/dL vs. 0.07 (0.04–0.11) mg/dL, *p* = 0.003]-No significant difference in FMD between the treatments [median (interquartile range): 3.1% (1.5–5.3%) vs. 3.3% (1.9–5.2%), *p* = 0.384]-[71]0.5 mg/day or no colchicine; Follow-up: a median of 3 years.Clinical trial with a prospective, randomized, observer-blinded endpoint design 532 patients with stable coronary disease receiving aspirin and/or clopidogrel (93%) and statins (95%)-Occurrence of primary outcome (composite incidence of acute coronary syndrome, out-of-hospital cardiac arrest, or noncardioembolic ischemic stroke) in 5.3% patients on colchicine and 16.0% patients assigned no colchicine (hazard ratio: 0.33; 95% confidence interval [CI] 0.18 to 0.59; *p* < 0.001)-Colchicine in addition to statins and other standard secondary prevention therapies appeared effective for the prevention of cardiovascular events in patients with stable coronary diseaseIntestinal intolerance[72]1 mg followed by 0.5 mg 1 hour later or no colchicine, 6 to 24 h prior to cardiac catheterizationRandomized controlled trial 40 ACS patients, 33 with stable CAD, and 10 controls-Significantly reduced transcoronary gradients of all 3 cytokines in ACS patients by 40% to 88% (*p* = 0.028, 0.032, and 0.032, for IL-1β, IL-18, and IL-6, respectively
[73]Oral colchicine (1 mg followed by 0.5 mg 1 h later) or no treatmentRandomized controlled trial 21 ACS patients compared with 9 untreated healthy controls-Markedly reduced intracellular and secreted levels of IL-1β compared with pre-treatment levels (*p* < 0.05 for both)-Significantly diminished pro-caspase-1 mRNA levels (by 57.7%) and secreted caspase-1 protein levels (by 30.2%) compared with untreated patients (*p* < 0.05 for both)-[74]Orally 1.5 mg or no treatmentRandomized controlled trial 12 patients with ACS on colchicine vs. 13 assigned to no treatment-Markedly reduced transcoronary levels of CCL2, CCL5, and CX3CL1 in patients with ACS (*p* < 0.05)-In vitro colchicine suppressed CCL2 gene expression in stimulated monocytes (*p* < 0.05)-- Reduced the intracellular concentration of all 3 chemokines (*p* < 0.01) and impaired monocyte chemotaxis (*p* < 0.05)-[75]Either 0.5 mg/day colchicine plus OMT or OMT alone; follow-up for 1 year.Prospective nonrandomized observational study of 80 patients with recent ACS (<1 month)-Significantly reduced LAPV (mean 15.9 mm3 [−40.9%] vs. 6.6 mm3 [−17.0%]; *p* = 0.008) and hsCRP (mean 1.10 mg/l [−37.3%] vs. 0.38 mg/L [−14.6%]; *p* < 0.001) versus controls-Comparable decrease in total atheroma volume (mean 42.3 mm3 vs. 26.4 mm3; *p* = 0.28) and LDL levels (mean 0.44 mmol/L vs. 0.49 mmol/L; *p* = 0.21) in both groups-[76]Either low-dose colchicine (0.5 mg once daily) or placeboRandomized, double-blind trial involving 4745 patients with fresh myocardial infarction (within 30 days form event)-The hazard ratios were 0.84 (95% CI, 0.46 to 1.52) for death from cardiovascular causes, 0.83 (95% CI, 0.25 to 2.73) for resuscitated cardiac arrest, 0.91 (95% CI, 0.68 to 1.21) for myocardial infarction, 0.26 (95% CI, 0.10 to 0.70) for stroke, and 0.50 (95% CI, 0.31 to 0.81) for urgent hospitalization for angina leading to coronary revascularization-Significantly lower risk of ischemic cardiovascular events compared to placeboPneumonia as a serious adverse event in 0.9% of the patients in the colchicine group vs. 0.4% of those in the placebo group (*p* = 0.03)[77]Colchicine or placebo within 30 days post-MIThe COLchicine Cardiovascular Outcomes Trial (COLCOT)-Significant reduction in the incidence of the primary endpoint for patients in whom colchicine was initiated < day 3 compared with placebo [HR = 0.52, 95% CI 0.32-0.84], in contrast to patients in whom colchicine was initiated between days 4 and 7 (HR = 0.96, 95% CI 0.53-1.75) or > day 8 (HR = 0.82, 95% CI 0.61–1.11).-Beneficial effects of early initiation of colchicine were seen for urgent hospitalization for angina requiring revascularization (HR = 0.35); all coronary revascularization (HR = 0.63); and the composite of cardiovascular death, resuscitated cardiac arrest, MI, or stroke (HR = 0.55, all *p* < 0.05).-[80].CanakinumabSubcutaneous placebo or canakinumab at doses of 5, 15, 50, or 150 mg monthly (follow-up: 4 months)Double-blind, multinational phase IIb trial of 556 patients with well-controlled diabetes mellitus and high cardiovascular risk -Modest but nonsignificant effects on the change in hemoglobin A1c, glucose, and insulin levels-Median reductions in C-reactive protein at 4 months were 36.4%, 53.0%, 64.6%, and 58.7% for the 5-, 15-, 50-, and 150-mg canakinumab doses, respectively, compared with 4.7% for placebo (all *p* ≤ 0.02)-Median reductions in interleukin-6 at 4 months across the canakinumab dose range tested were 23.9%, 32.5%, 47.9%, and 44.5%, respectively, compared with 2.9% for placebo (all *p* ≤ 0.008)-Median reductions in fibrinogen at 4 months were 4.9%, 11.7%, 18.5%, and 14.8%, respectively, compared with 0.4% for placebo (all *p* ≤ 0.0001)Clinical adverse events were similar in the canakinumab and placebo groups[101]50 mg, 150 mg, and 300 mg, administered subcutaneously every 3 months vs. placeboA randomized, double-blind trial involving 10,061 patients with previous myocardial infarction and a hs CRP level of 2 mg/L or more-Reduction in hsCRP (at 48 months): median 26% in the group receiving 50-mg dose, 37% in 150-mg group, and 41% in the 300-mg group compared to the placebo-Dose of 150 mg every 3 months led to a significantly lower rate of recurrent cardiovascular events (HR, 0.85 (95% CI, 0.74 to 0.98; *p* = 0.021)-The 150-mg dose met the prespecified multiplicity-adjusted threshold for statistical significance for the primary end point and the secondary end point (HR vs. placebo, 0.83; 95% CI, 0.73 to 0.95; *p* = 0.005)Higher incidence of fatal infection compared to placebo.[7]3 subcutaneous doses of canakinumab (50 mg, 150 mg, or 300 mg) once every 3 monthsCanakinumab Anti-Inflammatory Thrombosis Outcomes Study (CANTOS) 4833 stable atherosclerosis patientsIn patients with on-treatment IL-6 levels <1.65 ng/L:-32% reduction in MACE (HRadj) 0.68, 95% CI 0.56–0.82; *p* < 0.0001)-30% reduction in MACE plus the additional endpoint of hospitalization for unstable angina requiring urgent revascularization (MACE +, HRadj 0.70, 95% CI 0.59–0.84; *p* < 0.0001)-52% reduction in cardiovascular mortality (HRadj 0.48, 95% CI 0.34–0.68; *p* < 0.0001)-48% reduction in all-cause mortality (HRadj 0.52, 95% CI 0.40–0.68; *p* < 0.0001) with prolonged treatment.-Patients with on-treatment IL-6 levels ≥ 1.65 ng/L—no significant benefit for any of these endpointsNo related hepatic or renal toxicities Reduced rates of lung cancer increase in fatal infection [101]Canakinumab (150 mg subcutaneously) or placebo monthly for up to 12 monthsMulticenter, prospective, randomized, double-blind, placebo-controlled clinical trial involving outpatients with PAD and IC-Reduced markers of systemic inflammation (Il-6 and hsCRP) as early as 1 month after treatment-Lack of plaque progression alteration in the SFA, (however, early signal of improvement of maximum and pain-free walking distance in patients with symptomatic PAD)Safe and well tolerated[102]ZiltivekimabSubcutaneous administration of placebo or ziltivekimab 7.5 mg, 15 mg, or 30 mg every 4 weeks up to 24 weeksRESCUE, a randomised, double-blind, phase 2 trial carried out at 40 clinical sites in the USA. Participants with moderate to severe chronic kidney disease, and high-sensitivity CRP of at least 2 mg/L-median reduction in hsCRP levels: 77% for the 7.5 mg group, 88% for the 15 mg group, and 92% for the 30 mg group compared with 4% for the placebo group (all *p* < 0.0001)-Stable effects over the 24-week treatment period-Dose-dependent reductions in fibrinogen, serum amyloid A, haptoglobin, secretory phospholipase A2, and lipoprotein(a)Well tolerated, No serious injection-site reactions, sustained grade 3 or 4 neutropenia or thrombocytopenia.[104]LosmapimodLosmapimod 7.5 mg once daily (lower dose), twice daily (higher dose) or placebo for 84 days99 patients with atherosclerosis on stable statin therapy-Statistically significant reduction in average TBR in active segments (TBR ≥ 1.6) (HD vs. placebo: ΔTBR: −0.10 [95% CI: −0.19 to −0.02], *p* = 0.0125; LD vs. placebo: ΔTBR: −0.10 [95% CI: −0.18 to −0.02], *p* = 0.0194)-High dose group: decreased placebo-corrected inflammatory biomarkers including hsCRP (% reduction: −28% [95% CI: −46 to −5], *p* = 0.023) as well as FDG uptake in visceral fat (ΔSUV: −0.05 [95% CI: −0.09 to −0.01], *p* = 0.018), but not subcutaneous fa-[109]Oral losmapimod (7.5 mg or 15.0 mg loading dose followed by 7.5 mg twice daily) or matching placeboA double-blind, randomised, placebo-controlled trial of patients with NSTEMI-Decreased hsCRP concentrations at 72 h in the losmapimod group compared to the placebo group (geometric mean 64.1 nmol/L, 95% CI 53.0–77.6 vs 110.8 nmol/L, 83.1–147.7; *p* = 0.0009)-Significantly lower geometric mean BNP concentrations at 12 weeks (37.2 ng/L, 95% CI 32.3–42.9 vs 49.4 ng/L, 38.7–63·0; *p* = 0.04)Safety outcomes did not differ between groups[110]Either twice-daily losmapimod (7.5 mg) or matching placebo on a background of guideline-recommended therapy.LATITUDE-TIMI 60, a randomized, placebo-controlled, double-blind, parallel-group trial conducted at 322 sites in 34 countries 3503 participants (part A)Among patients with acute MI, use of losmapimod compared with placebo did not reduce the risk of major ischemic cardiovascular events (primary end point occurrence by 12 weeks: placebo (7.0%) and patients treated with losmapimod: 8.1%; hazard ratio, 1.16; 95% CI, 0.91–1.47; *p* = 0.24Similar on-treatment rates of serious adverse events: 16.0% with losmapimod and 14.2% with placebo[111]MK2206Injection of MK2206 at a dose of 4 mg/kg/d, or equal volume of saline (containing 0.1% DMSO)In vitro, animal study-Significant reduction in vascular inflammation, atherosclerotic lesions, and inhibition of proliferation of VSCM in ApoE^−/−^ mice in vivo-Diminished lipid accumulation (associated with the enhanced cholesterol efflux)-Decreased inflammatory response by modulating inflammation-related mRNA stability in macrophages-Suppression of migration, proliferation, and inflammation in VSCMs-Inhibition of proliferation and inflammation of endothelial cells-[112]
Cultured human hepatoma cells-Stimulated proteolytic processing of SREBP-2-Upregulation of LDLR expression-Stimulation of LDL uptake-[115]Inclacumab1 infusion of placebo or inclacumab (5 or 20 mg/kg, administered between 1 and 24 h before PCI)The Effects of the P-Selectin Antagonist Inclacumab on Myocardial Damage After Percutaneous Coronary Intervention for Non-ST-Segment Elevation Myocardial Infarction (SELECT-ACS) 544 patientsPatients receiving inclacumab 20 mg/kg with a short time interval between infusion and PCI:
-placebo-adjusted geometric mean percent changes in troponin I, creatine kinase-myocardial band, and peak troponin I at 24 h were −45.6% (*p* = 0.005), −30.7% (*p* = 0.01), and −37.3% (*p* = 0.02), respectively
Patients with a long time interval between infusion and PCI: no significant changes were observed in
-Dose of 20 mg/kg significantly reduces myocardial damage after PCI in NSTEMI patients and benefits are greater when the infusion is administered <3 h before PCI
-[145]Each dose level (0.03–20 mg/kg) investigated in separate groups of 8 subjects (6 on inclacumab, 2 on placebo)Randomized, double-blind placebo-controlled study 56 healthy subjects-Inclacumab was well tolerated Most common AEs: headache, cough, sore throat, and upper respiratory tract infection, one serious AE (rhabdomyolysis)[142]
ABC—ATP-binding cassette; ABCA1—ATP-binding cassette transporters A1; ABCG1—ATP-binding cassette transporters G1; ACS—acute coronary syndrome; CAD—coronary artery disease CC—cholesterol crystals; CI—confidence intervals ; FH—familiar hypercholesterolemia; HDL—high-density lipoprotein; HR—hazard ratios; HRadj—multivariable adjusted hazard ratio; IC—intermittent claudication; LAPV—low attenuation plaque volume; LDL—low-density lipoprotein; LXR—liver X receptor; MACE—major adverse cardiovascular events; NSTEMI—non-ST-segment elevation myocardial infarction; OMT—optimal medical therapy; PAD—peripheral artery disease; ROS—reactive oxygen species; TBR—tissue-to-background ratio; TG—triglyceride; VLDL—very-low-density lipoprotein cholesterol; VSCM—vascular smooth muscle cell.


## 12. Conclusions

The shift of the target from lipid metabolism has opened the door to many new therapeutic targets. Currently, most of the known targets for anti-atherosclerotic drugs not only focus on lipid storage disease but also on inflammation (which is a common mediator of many risk factors), as well as mechanisms of innate and adaptive immunity in atherosclerosis, molecule scavengers, etc. The success of targeting inflammatory agents (e.g., IL-1β) confirms that the inflammasome pathway may pose a promising pathway for further interventions. The therapeutic potential of cyclodextrins, protein kinase inhibitors, colchicine, inhibitors of p38 mitogen-activated protein kinase (MAPK), lipid dicarbonyl scavengers, a monoclonal antibody targeting interleukin-1β, and P-selectin inhibitors is still not fully confirmed and requires confirmation in large clinical trials. The preliminary results look promising. However, there is a need for reliable biomarkers which could enable the determination of whether the interventions are efficient as well as the monitoring of atherosclerosis progress cessation of regression. Furthermore, a better understanding of the mechanism underlying the effects of such drugs will help to alleviate or eliminate adverse effects or establish a group of patients who would benefit most from the treatment. Moreover, it appears that a combination of treatment targeted at different pathways involved in the development of atherosclerosis may be more beneficial than a single drug due to the fact that this disease is multifactorial.

## Data Availability

Not applicable.
